# A novel porcine model reproduces human oculocutaneous albinism type II

**DOI:** 10.1038/s41421-019-0117-7

**Published:** 2019-10-08

**Authors:** Ying Zhang, Qianlong Hong, Chunwei Cao, Lizhu Yang, Yongshun Li, Tang Hai, Hongyong Zhang, Qi Zhou, Ruifang Sui, Jianguo Zhao

**Affiliations:** 1 State Key Laboratory of Stem Cell and Reproductive Biology, Institute of Zoology, Chinese Academy of Sciences, 100101 Beijing, China; 2 University of Chinese Academy of Sciences, 100049 Beijing, China; 3 School of Life Sciences, Anhui University, 230601 Hefei, China; 4 Department of Ophthalmology, Peking Union Medical College Hospital, Peking Union Medical College, Chinese Academy of Medical Sciences, 100730 Beijing, China

**Keywords:** Mechanisms of disease, Developmental biology

Dear Editor,

Oculocutaneous albinism (OCA) represents a genetically heterogeneous group of disorders characterized by absent or reduced pigmentation of the skin, hair, and eyes from the time of birth^1^. OCA type II (OCA2) is one of the most common type of the disorder, and accounts for 30% of cases worldwide^2^. However, no effective treatments or medicines exist for curing this disease currently, thus it is necessary to generate animal models for evaluating novel medicines or developing novel therapeutic interventions for the clinic. Previous studies have reported several mouse models for OCA2. However, in addition to showing some of the clinical manifestations of OCA2, several mutant murine strains are accompanied by other abnormalities, including decreased neonatal viability, increased prenatal lethality, reproductive and neurological disorders, and incidence of cleft palate^3,4^. It suggests that mice may not fully recapitulate the OCA phenotype, thus highlighting the need for a more suitable animal model. Here, we created a porcine model of OCA2 to bridge the gap between human clinical cases and rodent animal models, and the porcine model displays overt hypopigmentation in eyes and hair follicles similar to those observed in OCA2 patients and lacked other apparent abnormities.

Compared to the coat color pattern of a unique two-end black, the mutant Bama miniature pigs in the Z0015 line exhibiting a two-end diluted brown coat color were identified from an ethylnitrosourea (ENU) mutagenesis program^5^ (Fig. 1a). Pink eyes also accompanied the dilution of coat color in the mutant pigs (Fig. 1b); these unique features were similar to those of OCA in patients^6^. In the mutant pig line, a G1 boar (Z0015) was backcrossed to four G2 sows that produced 65 G3 offspring. Of the progeny, 21.5% (8 males and 6 females) exhibited the mutant phenotype in an autosomal recessive inheritance pattern (Fig. 1c, d).

**Fig. 1:**
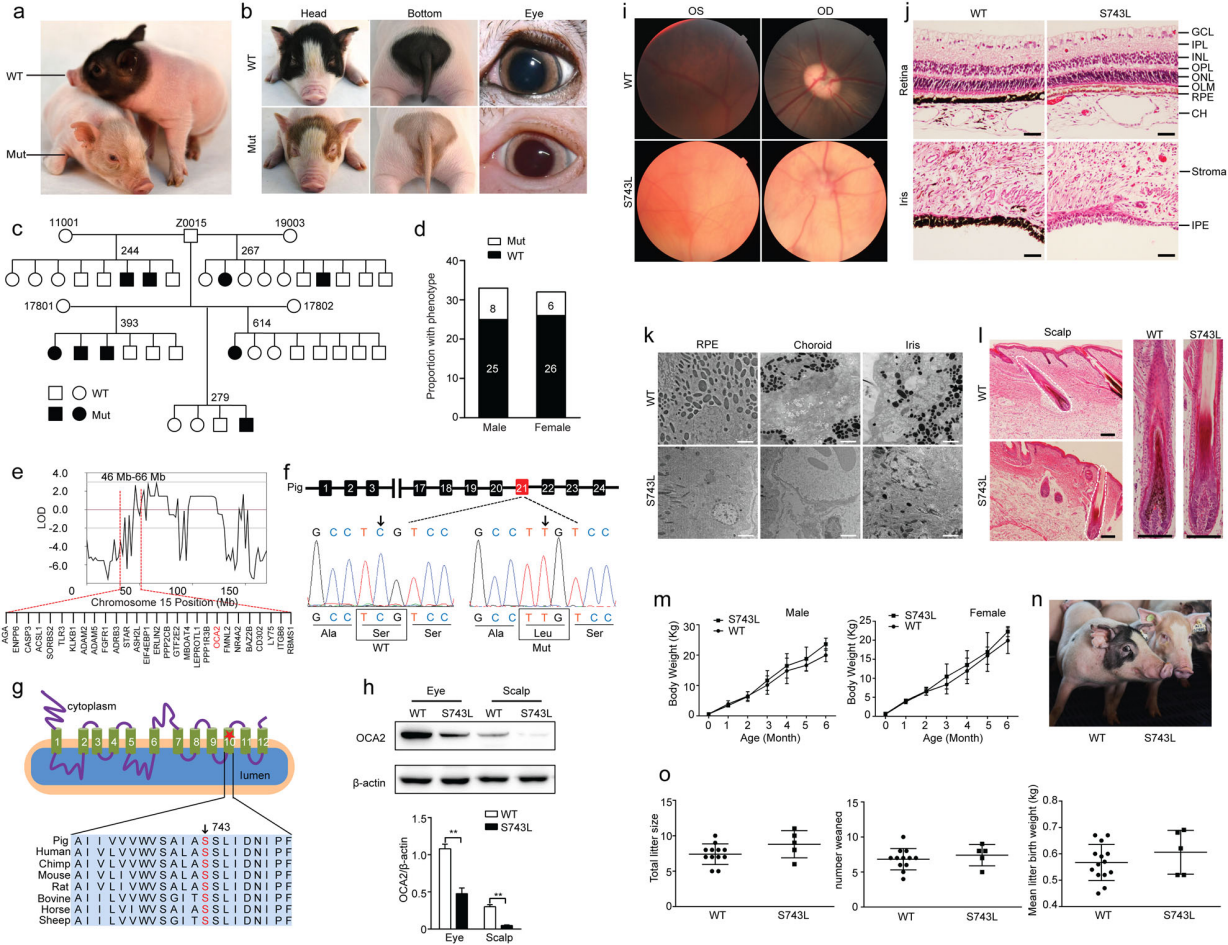
A pig model of OCA2 resulted from the S743L mutation in the *OCA2* gene **a** An 11-day-old mutant exhibits a significant dilution of coat color compared with its littermate control. **b** The mutant exhibits two-end diluted brown coat color with pink eyes. **c** The pedigree of the mutant family and **d** mutant G3 animals exhibiting an autosomal recessive mode of inheritance. WT: wild-type; Mut: mutant. **e** A significant linkage peak between 46 Mb and 66 Mb on chromosome 15 (LOD > 2) containing 30 annotated genes was identified. **f** The mutation c.2228 C > T was located in exon 21 and resulted in a change of serine to leucine at position 743 (S743L). The arrows indicate the position of the missense mutation. **g** The S743L (red star) in the transmembrane domain 10 and the serine residue (arrow) in the S743L variant is highly conserved in mammalian OCA2 proteins. **h** The protein expression of OCA2 from mutants was significantly lower than that from WT pigs in eyes and scalp (WT: *n* = 3, S743: *n* = 3; **P < 0.01). **i** Fundus photographs of S743L p**i**gs showed overt hypopigmentation in both eyes compared with WT pigs. OS (*oculus sinister*): left eye; OD *(oculus dexter*): right eye. **j** The ocular tissues from S743L pigs revealed an obvious reduction in melanin in RPE cells, choroid (CH), and IPE cells compared with those from WT pigs. OLM, outer limiting membrane; ONL, outer nuclear layer; OPL, outer plexiform layer; INL, inner nuclear layer; IPL, inner plexiform layer; GCL, ganglion-cell layer. **k** The number of pigmented melanosomes decreased in the iris, RPE, and choroid of S743L pigs compared with that in WT pigs. **l** The S743L pigs displayed a reduction of melanin in hair follicles. The right panel indicates a magnification of hair follicle in the dashed line. Scale bars: 50 μm. **m** The male and female S743L pigs exhibited a similar growth curve to those of age-matched WT piglets over a 6-month period (male WT: *n* = 20, S743: *n* = 8; female WT: *n* = 20, S743: *n* = 6; *P* > 0.05). **n** A 6-month-old mutant exhibits a dilution of coat color compared with its littermate control. **o** The reproductive traits of S743L pigs were indistinguishable from those of WT piglets, including total litter size, number weaned, and mean litter birth weight (WT: *n* = 12, S743: *n* = 5; *P* > 0.05)

To identify the causative gene in the Z0015 line, we performed a family-based genome-wide linkage study (GWLS) to map the chromosome regions co-segregating with the mutant phenotypes. A significant linkage peak was identified between 46 Mb and 66 Mb on chromosome 15 (LOD > 2) containing 30 annotated genes (Fig. 1e). Of these candidate genes, *OCA2* was of particular interest due to pathogenic mutations in the *OCA2* gene are known to cause OCA2, one subtype of OCA^7^. Using genomic DNA, 23 coding exons flanked by introns of the porcine *OCA2* gene (GenBank ID: 397171) were screened. The results showed a single missense mutation c.2228 C > T that resulted in a serine transition to leucine (S743L, hereafter referred to as *OCA2*^*S743L/S743L*^) in exon 21 (Fig. 1f).

To further investigate the association between the altered allele and the mutant phenotype, we genotyped the c.2228 C > T mutation in various pig breeds and the results revealed that the T/T homozygous mutant genotype was only found in the affected pigs from the Z0015 line but not in the other Bama miniature pigs or other breeds (Supplementary Table S1). Together, the above results suggest that the c.2228 C > T mutation in *OCA2* co-segregated with the OCA phenotype and is the causative mutation in this mutant line. In addition, the S743L mutation in OCA2 was not found in the pig SNPs database, suggesting that S743L was induced by ENU mutagenesis.

The S743L mutation is located in the predicted transmembrane domain 10 of the porcine OCA2 protein and is evolutionarily conserved among distinct mammals (Fig. 1g), indicating its vital role within this domain for the function of *OCA2*. The S743L mutation did not alter *OCA2* expression at the transcript level (Supplementary Fig. S1); however, the protein expression from mutant pigs was significantly lower than that from WT pigs in eyes and scalp (Fig. 1h). Furthermore, supporting our discovery, the patient manifests a typical moderate OCA2 phenotype was found carrying a compound heterozygote for two missense substitutions, S736L (orthologous mutation to porcine S743L) and G27R^8^. This suggests that S743L-mutant pigs are suitable for modeling human OCA2 due to uniform genetics.

We further analyzed the morphological and pathological phenotypes in the eyes and skin of the *OCA2*^*S743L/S743L*^ pigs. Fundus photographs showed overt hypopigmentation in both eyes from the mutants compared with the unaffected controls (Fig. 1i). The ocular tissues from mutant pigs revealed an obvious reduction of melanin in the retinal pigment epithelium (RPE) cells and choroid (CH), and an almost complete lack of melanin in the iris pigment epithelium (IPE) cells (Fig. 1j). Furthermore, iris, RPE, and CH of mutants displayed an evident decrease in the number of the pigmented melanosomes compared to those of the WTs, which was consistent with the histopathological findings (Fig. 1k). Electroretinograms (ERGs) were used to assess changes in retinal function, and the results showed that no significant functional differences existed between mutant and WT pigs (Supplementary Fig. S2). In addition, the mutants also exhibited decreased levels of melanin in scalp hair follicles compared with those from WT pigs (Fig. 1l). Together, these phenotypes manifest the clinical features of human OCA2^9^.

To investigate whether the S743L mutation induced growth or fertility aberrations in *OCA2*^*S743L/S743L*^ pigs, we characterized the growth and reproductive traits. The growth curve revealed that the body weight of *OCA2*^*S743L/S743L*^ piglets was similar to that of the age-matched WT piglets over a period of 6 months (Fig. 1m). The adult mutant pigs showed a consistent dilution brown coat color and pink eyes, and their physique was similar to that of WT pigs (Fig. 1n). At 6 months or greater, *OCA2*^*S743L/S743L*^ males were mated with *OCA2*^*S743L/S743L*^ females and shown to be fertile. The reproductive traits of *OCA2*^*S743L/S743L*^ pigs were indistinguishable from those of WT piglets (Fig. 1o). In addition, the profiles of blood physiological and biochemical parameters of *OCA2*^*S743L/S743L*^ pigs at 3 months were consistent with those of age-matched WT pigs (Supplementary Fig. S3). Our results indicate that while the *OCA2*^*S743L/S743L*^ mutation resulted in OCA disease phenotype but did not affect growth, reproduction, and levels of blood physiology and biochemistry.

In summary, we identified a first pig model of OCA2 resulted from the S743L mutation in the *OCA2* gene. Furthermore, the S743L mutation, which is orthologous to the human S736L mutation, results solely in pigmentation-related defects without other apparent abnormities similar to those observed in OCA2 patients. Taken together, our findings suggested that *OCA2*^*S743L/S743L*^ pigs could be utilized as an appropriate preclinical model in which to test human-size interventional devices and optimize candidate therapies before advancing to clinical trials, thus accelerating the development of effective applications for OCA patients.

## Supplementary information

Supplementary Information.

## Supplementary information

**Supplementary Information** accompanies the paper at (10.1038/s41421-019-0117-7).
