# Supplementary material for: A novel porcine model reproduces human oculocutaneous albinism type II
Source: Cell Discov. 2019 Oct 8;5:48. doi: 10.1038/s41421-019-0117-7 (PMC6796836; doi:10.1038/s41421-019-0117-7)
Supplement: Supplementary file 1 — Supplementary Information. [file 41421_2019_117_MOESM1_ESM.pdf]

Supplementary Information

Materials and methods

Pigs

The Bama miniature pigs were raised at the Beijing Farm Animal Research Center (attached to the Institute of Zoology, Chinese Academy of Sciences) and had ad libitum access to a commercial pig diet (nutrient levels based on the United States National Research Council recommendations) and water throughout the experiment. The founder Bama miniature pigs carrying the S743L mutation were generated in a large-scale ENU mutagenesis screen [1]. All experiments involving pigs were approved by the Institutional Animal Care and Use Committee of the Institute of Zoology, Chinese Academy of Sciences, China.

GWLS and genotyping

Genomic DNA was isolated from the ear tissues using phenol/chloroform extraction. In total, nine mutant and twelve wild-type (WT) animals were genotyped using porcine SNP60 BeadChips for the GWLS analysis. Raw data were processed by removing SNPs with a call rate <90% and a minor allele frequency (MAF) <0.05. Then, Merlin software was used to perform genome-wide and family-based linkage analysis. In addition, parametric linkage analysis assuming a recessive model was conducted, and the LOD score was calculated to assess evidence for linkage.

For the candidate gene OCA2, primers were designed to allow amplification of 23 encoding exons from ear genomic DNA used as templates in 9 affected and 6 WT pigs, and PCR products were analyzed by Sanger sequencing. Primers are listed in Supplementary Table S2.

qRT-PCR

Total RNA was isolated from tissues using Trizol (Thermo Fisher Scientific Inc., Carlsbad, CA, USA) and reverse transcribed to cDNA using a FastQuant RT kit (Tiangen Bio Co., Ltd, Beijing, China). qRT-PCR was performed on a Stratagene Mx3005P real-time PCR system (Agilent Technologies Inc., Santa Clara, CA, USA) using a SYBR Premix EX Taq kit (Takara Bio Inc., Otsu, Shiga, Japan). The relative mRNA expression levels of the target genes were calculated as the fold changes of the threshold cycle (Ct) value relative to the reference using the $2^{-\Delta\Delta Ct}$ method. The sequences of the primers used are listed in Supplementary Table S2.

Western blot

Tissues were lysed in RIPA lysis buffer (CWBIO Co., Ltd., Beijing, China) and supplemented with protease inhibitor cocktail (Thermo Fisher Scientific Inc., Rockford, IL, USA). Lysates were mixed with an equal volume of 2x Laemmli sample buffer (Bio-Rad Laboratories Inc., Hercules, CA, USA) and boiled for 5 minutes. The protein extracts were electrophoresed on 12% SDS-PAGE gels and were transferred to PVDF membranes (Bio-Rad Laboratories Inc., Hercules, CA, USA). Membranes were blocked with 5% skimmed milk for 2 h at room temperature and incubated overnight at 4 °C with primary antibodies against OCA2 (Novus Biologicals, Inc., Littleton, CO, USA, 1:1,000 dilution) and β-actin (Sungene Biotech Co., Ltd, Tianjin, China, 1:10,000 dilution). The detection was carried out with horseradish peroxidase–conjugated (HRP) goat anti-rabbit or mouse secondary antibody (Sungene Biotech Co., Ltd, Tianjin, China, 1:10,000 dilution) and visualized with an enhanced chemoluminescence (ECL) kit (Thermo Fisher Scientific Inc., Rockford, IL, USA), according to the manufacturer's

protocol.

Fundus photographs

The pigs were anaesthetized with Zoletil 50 (0.1 mL/kg, Virbac, Carros, France), and pupils were dilated with tropicamide and phenylephrine hydrochloride (Santen Pharmaceutical Co., Ltd., Japan). Fundus photographs were taken with a retinal fundus camera (TRC-NW6S, Topcon Co., Tokyo, Japan).

Histopathology

Tissues were fixed with 4% neutral buffered paraformaldehyde (PFA). The fixed tissues were embedded in paraffin according to standard laboratory procedures [2]. Five micrometer-thick sections of paraffin-embedded tissues were processed for H&E staining.

Transmission electron microscopy

The porcine eyes were fixed with 2.5% glutaraldehyde in 0.2 M cacodylate buffer overnight. After washing in 0.2 M PB, the tissue was cut into small pieces, approximately 1 mm^3, and immersed in 1% OsO4 in 0.2 M cacodylate buffer for 2 h at 4 °C. Then, the samples were dehydrated through a graded ethanol series and embedded in resin. Ultrathin sections were cut on a Leica EM UC6 ultramicrotome (Leica Microsystems Inc., Wetzlar, Germany), and stained with uranyl acetate and lead citrate. The morphology of the samples was characterized with aFEI Tecnai G2 F20 transmission electron microscope (FEI Co., Eindhoven, Netherlands) at 200 kV.

ERGs

Animals were anaesthetized with Zoletil 50 (0.1 mL/kg, Virbac, Carros, France) and pupils were dilated with tropicamide and phenylephrine hydrochloride (Santen Pharmaceutical Co., Ltd., Japan). Before ERGs recordings, dark adaptation was provided for at least 30 min. Binocular stimulation was used in all ERG recordings. ERGs were recorded with a RETI-Port 32 system (Roland Consult, Wiesbaden, Germany) in accordance with International Society for Clinical Electrophysiology of Vision standards. Scotopic ERGs were obtained by averaging 3 responses to −25-dB flashes of 0.5 Hz against a dark background of a Ganzfeld screen. The bandpass filter of the amplifier was set to 1–300 Hz. Photopic ERGs were obtained by averaging 3 responses to a 0-dB standard flash stimulation of 0.5 Hz with the background light on. The bandpass filter was set to 1–300 Hz.

Measurements of physiological and biochemical parameters

Blood samples were obtained by puncturing the precaval vein of 3-month-old pigs that had been fasted overnight. The physiological parameters were measured in EDTA-K2-treated blood using a RAYTO RT-7600S automatic hematology analyzer (Rayto Life and Analytical Sciences Co., Ltd., Guangzhou, China). Plasma from clot activator-treated blood was separated by refrigerated centrifugation at 1000 *g* for 5 minutes and was analyzed using a Hitachi 7080 automatic biochemical analyzer (Hitachi Instrument Inc., Tokyo, Japan) with adapted reagents (Chemclin Biotech Co., Ltd., Beijing, China).

Statistical analysis

An unpaired 2-tailed Student's t test (for 2 groups) was applied to parametric data using GraphPad Prism software (GraphPad Inc., San Diego, CA, USA) or SPSS software (SPSS Inc., Chicago, IL, USA). Otherwise, the Mann-Whitney *U* test was applied for nonparametric data. The results are presented as the means ± SD unless otherwise indicated. A *P* value of < 0.05 was considered statistically significant.

References

1 Hai, T. *et al.* Pilot study of large-scale production of mutant pigs by ENU mutagenesis. *Elife*, 6: e26248, doi:10.7554/eLife.26248 (2017).

2 Zhang, Y. *et al.* Thyroid hormone regulates hematopoiesis via the TR-KLF9 axis. *Blood* **130**, 2161-2170, doi:10.1182/blood-2017-05-783043 (2017).

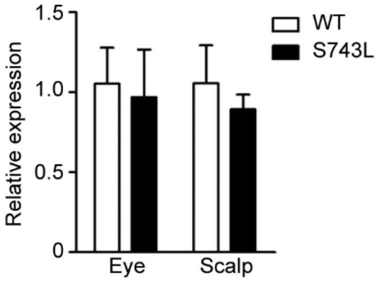

Supplementary Fig. S1 The S743L mutation does not alter OCA2 expression in eyes and scalp at the transcript level as determined with qRT-PCR (WT: n=6, S743: n=6; P>0.05).

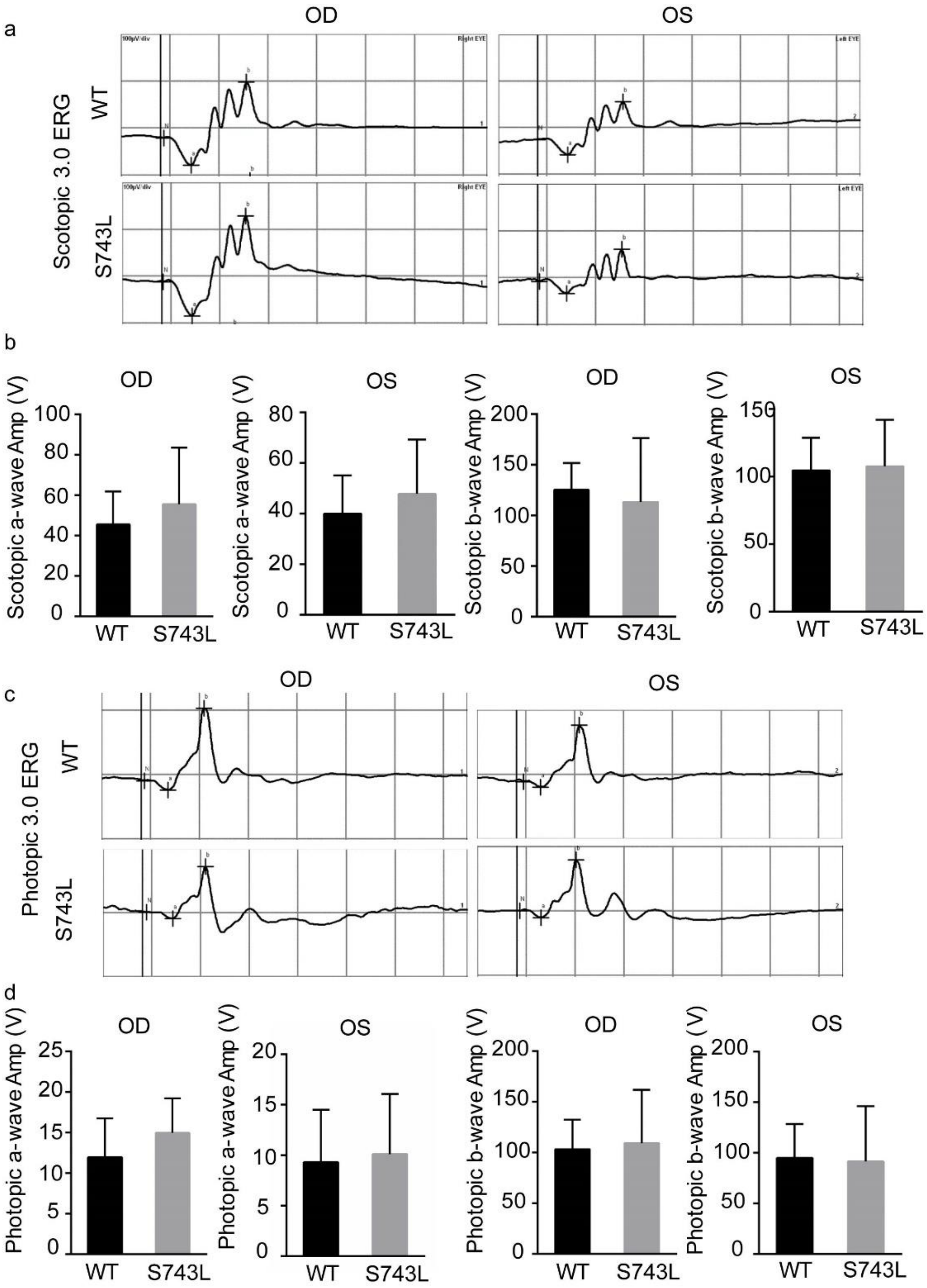

Supplementary Fig. S2 Electroretinograms (ERGs) were used to assess changes in retinal function. (a-b) No significant differences were found between mutant and WT pigs in scotopic ERGs. (c-d) No significant differences were found between mutant and WT pigs in photopic ERGs. (WT: n=3, S743: n=3; P>0.05).

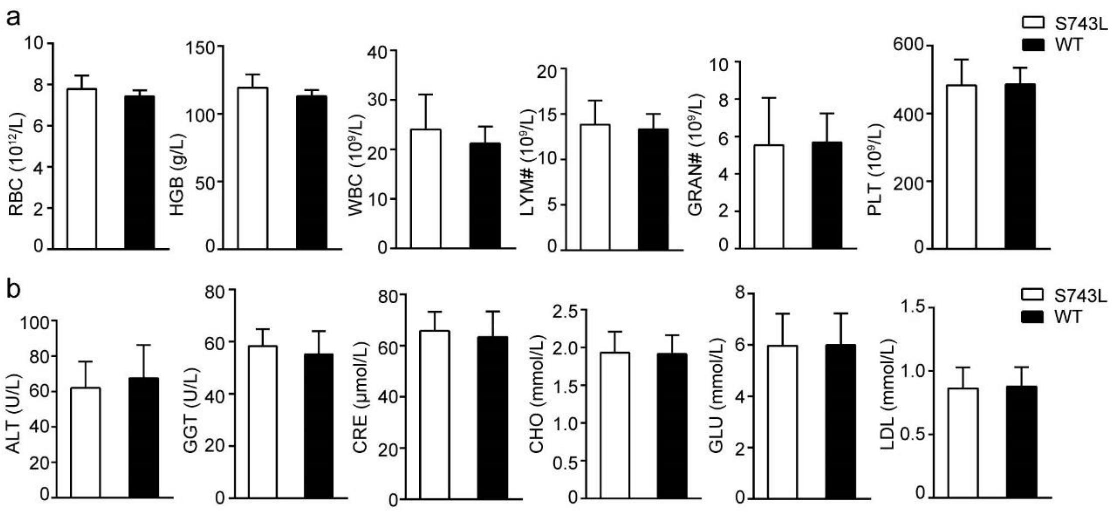

Supplementary Fig. S3 (a) At three months, blood physiological parameters of S743L pigs showed no significant differences compared with those of WT pigs (WT: n=15, S743: n=11; P>0.05). (b) At three months, blood biochemical parameters of S743L pigs showed no significant differences compared with those of WT pigs (WT: n=15, S743: n=11; P>0.05).

Supplementary Table S1. Genotyping results of the mutant Bama miniature pig line and various pig breeds.

Group	Phenotype	Number	CC	CT	TT	C%	T%
Z0015 Pedigree	Mutant	14	0	0	14	57.7	42.3
	WT	51	24	27	0		
Additional Pedigrees	WT	134	134	0	0	100	0
Large White	WT	30	30	0	0	100	0
Landrace	WT	30	30	0	0	100	0

Supplementary Table S2. List of primer sequences.

Name	Sequence (5'-3')	Usage
OCA2-21F	TGGTTGGCTTGAGTATGG	Genotyping
OCA2-21R	TTCTTTGAAGGAAGGGTG	Genotyping
cOCA2-F	CGGGACACTGATTGGAGCAT	qRT-PCR
cOCA2-R	ACCATCATTGGGAAGCCCAG	qRT-PCR
